# Delivery and postnatal care among women in 71 low- and middle-income countries: analyzing coverage gaps using household surveys

**DOI:** 10.1186/s12884-024-06681-y

**Published:** 2024-07-26

**Authors:** Emily B. Wilson, Lori Niehaus, Safia S. Jiwani, Elizabeth A. Hazel, Abdoulaye Maïga, Agbessi Amouzou

**Affiliations:** Johns Hopkins University Bloomberg School of Public Health, Baltimore, MD USA

**Keywords:** Maternal health, Household surveys, Coverage

## Abstract

**Background:**

High levels of maternal morbidity and mortality persist in low- and middle-income countries, despite increases in coverage of facility delivery and skilled assistance at delivery. We compared levels of facility birth to a summary delivery care measure and quantified gaps.

**Methods:**

We approximated a delivery care score from type of delivery (home, lower-level facility, or hospital), skilled attendant at delivery, a stay of 24-or-more-hours after delivery, and a health check within 48-h after delivery. Data were obtained from 333,316 women aged 15–49 who had a live birth in the previous 2 years, and from 71 countries with nationally representative surveys between 2013 and 2020. We computed facility delivery and delivery care coverage estimates to assess the gap. We stratified the analysis by country characteristics, including the national maternal mortality ratio (MMR), to assess the size of coverage gaps, and we assessed missed opportunities through coverage cascades. We looked at the association between MMR and delivery care coverage.

**Results:**

Delivery care coverage varied by country, ranging from 24% in Sudan to 100% in Cuba. Median coverage was 70% with an interquartile range of 30 percentage points (55% and 85%). The cascade showed that while 76% of women delivered in a facility, only 41% received all four interventions. Coverage gaps exist across all MMR levels. Gaps between highest and lowest wealth quintiles were greatest in countries with MMR levels of 100 or higher, and the gap narrowed in countries with MMR levels below 100. The delivery care indicator had a negative association with MMR.

**Conclusions:**

In addition to providing high-quality evidenced-based care to women during birth and the postpartum period, there is also a need to address gaps in delivery care, which occur within and between countries, wealth quintiles, and MMR phases.

**Supplementary Information:**

The online version contains supplementary material available at 10.1186/s12884-024-06681-y.

## Background

A Sustainable Development Goal target is to reduce the global maternal mortality ratio (MMR) to less than 70 maternal deaths per 100,000 live births by 2030 [1]. In 2020, an estimated 223 maternal deaths per 100,000 live births occurred, 70% of which were in Sub-Saharan Africa [1]. Although maternal mortality has generally been in decline globally [2], women continue to die from complications due to pregnancy and childbirth [3].

Many maternal deaths could be averted through the provision of quality antenatal, delivery, and postnatal interventions [4]. The monitoring of delivery outcomes relies mostly on indicators that reflect women’s contacts with the health system rather than the quality of the care women received [5]. Effective coverage measures are needed to ensure quality care is received and individuals are experiencing health gains from a service [6].

There have been advances in the measurement of effective coverage of maternal and newborn health care in recent years [7–11], including innovative methodological approaches which link household and facility surveys [12, 13]. However, much work remains to be done. A comprehensive effective coverage measurement review found that pre-pregnancy, birth, and postnatal care interventions were the least documented in the literature [6]. Few population-based quality measures have been developed for delivery and postnatal care. Questions to reliably capture content of interventions received by women during childbirth and the postnatal period are still developing since limited questions which can approximate measures of quality-adjusted care have been collected as part of national surveys such as the Demographic and Health Surveys (DHS) or the Multiple Indicator Cluster Surveys (MICS). The Demographic and Health Survey program now includes questions in its core questionnaires to capture content of postnatal care by asking women to report whether their blood pressure was measured, or whether a health provider discussed vaginal bleeding and family planning with them postpartum [14].

With limited data on quality of delivery care, additional approaches are needed to assess the care that women are receiving. While the number of contact indicators does not speak directly to the quality of care women received, women who receive high quality care will have received all delivery and postnatal care contact interventions and co-coverage has been used as a proxy for measuring care quality and gaps [5, 15]. Summary indices are important to quality-of-care efforts because these measures show what percentage of the population is receiving all, or most, health interventions [8]. If women receive few essential interventions during childbirth and the postpartum period, efforts to improve the quality of care they receive will not have the intended effect.

Our aims were to develop a summary measure for delivery care and to estimate the gaps between facility delivery and delivery care coverage among women in LMICs by country, wealth quintile and MMR phase.

## Methods

### Data

Demographic and Health Surveys (DHS) [16] and Multiple Indicator Cluster Surveys (MICS) [17] are nationally representative household surveys that provide data on a wide range of population, health, and nutrition indicators, and which have been used for monitoring, evaluation, reporting, and research [18–20]. For this study we reviewed DHS and MICS surveys from 2013, when the postnatal-health check timing data became consistently available, to 2020 when we began data analysis. We excluded 12 surveys due to unavailable data sets or interview question responses. We dropped one cluster in South Africa and six clusters in India, which had only one primary sampling after stratification. Senegal’s continuous surveys in 2017, 2018, and 2019 were combined as one.

We analyzed 34 DHSs and 37 MICSs. The median survey year was 2017. Altogether, this study included a total of 333,316 women aged 15–49 whose most recent birth occurred within the two years prior to survey data collection. Country samples ranged from 321 women in the Congo to 86341 women in India.

### Delivery care measure

We reviewed the delivery and postnatal interventions for which data are systematically collected within DHS and MICS. We then constructed a summary delivery care measure by combining the four maternal health interventions which are standard within these surveys: delivery location (home, hospital, or other health facility), skilled attendant at birth, facility stay of at least 24 h after delivery, and postnatal health check within forty-eight hours following childbirth. Facility delivery type distinguished between delivery in a hospital versus lower-level facility. Combined private hospitals and clinics were considered as hospital delivery. Skilled birth attendant was defined based on the country definition included in each survey. Skilled attendant and facility categorizations can be found in Supplementary Files 1 and 2, respectively. Facility stay after delivery distinguished less than 24 h from 24 + hours, defined according to the World Health Organization’s recommendation [21]. Postnatal care used a forty-eight-hour cut-off based on the standard international indicator which is defined as the “postnatal health check for the mother (or newborn) within two days of delivery” [22]. These four indicators encompass the continuum of maternal care, recommended for all women who give birth, for which data are widely available.

Table 1 shows each indicator, respondent categories, and the associated score. We used an additive approach by summing each birth’s total score. Scores ranged from 0 to 5. Following the literature [23], to avoid many missing values for the delivery care score in which one of the component indicators was not answered by some women, missing data was imputed as zero. A woman was assumed not to have received interventions for which there was no response. Scores were weighted, rescaled 0–100%, and used as an approximation of quality-adjusted coverage. Zero indicates low quality delivery care while 100 approximates high quality delivery care.

**Table 1:** Indicators and scores included in the definition of the delivery care indicator

Indicator	Respondent category	Score
Facility delivery type	Hospital	2
	Lower lever	1
	Home	0
Skilled attendant at birth	Skilled attendant	1
	Unskilled attendant	0
Facility stay	24 + hours	1
	< 24 h	0
First postnatal care	< 48 h after delivery	1
	> 48 h after delivery	0
	None	0

### Statistical analysis

We reported weighted percentages of women who received all standard and delivery care interventions for each survey, and the pooled sample of all surveys. For the pooled sample, we used the inverse proportion of women, contributed by each country, as an additional adjustment weight to account for different sample sizes across countries. We also reported the median and interquartile range for country delivery care estimates.

We constructed effective coverage cascades following the recommendation of Amouzou and colleagues [6]. The cascade includes 1) the population in need: women whose most recent birth was in the two years preceding the survey; 2) women who delivered in a facility; 3) women who delivered in a facility with a skilled attendant; 4) women who delivered in a facility, with a skilled attendant, and stayed in the facility for 24 + hours; and 5) women who delivered in a facility, with a skilled attendant, who stayed in the facility for 24 + hours, and had a health check within 48 h postpartum. Pooled data cascades are reported by facility type and by maternal mortality ratios (MMR) [24, 25] grouped according to obstetric transition model phase [26]. Country level cascades are reported by obstetric transition model phase.

We assessed delivery care coverage between the highest and lowest wealth quintile and reported the size of the gap in each MMR phase. We also reported the difference between delivery care coverage and facility delivery coverage in each country. We analyzed the linear association between MMR and the delivery care indicator with the hypothesis that there is a negative linear association.

All data are publicly available. Analyses were conducted in R version 4.2.2 [27]. All analysis code is publicly available on the Countdown to 2030 Github repository:https://github.com/countdownto2030/delivery_care_coverage_gaps.

## Results

**Fig. 1:**
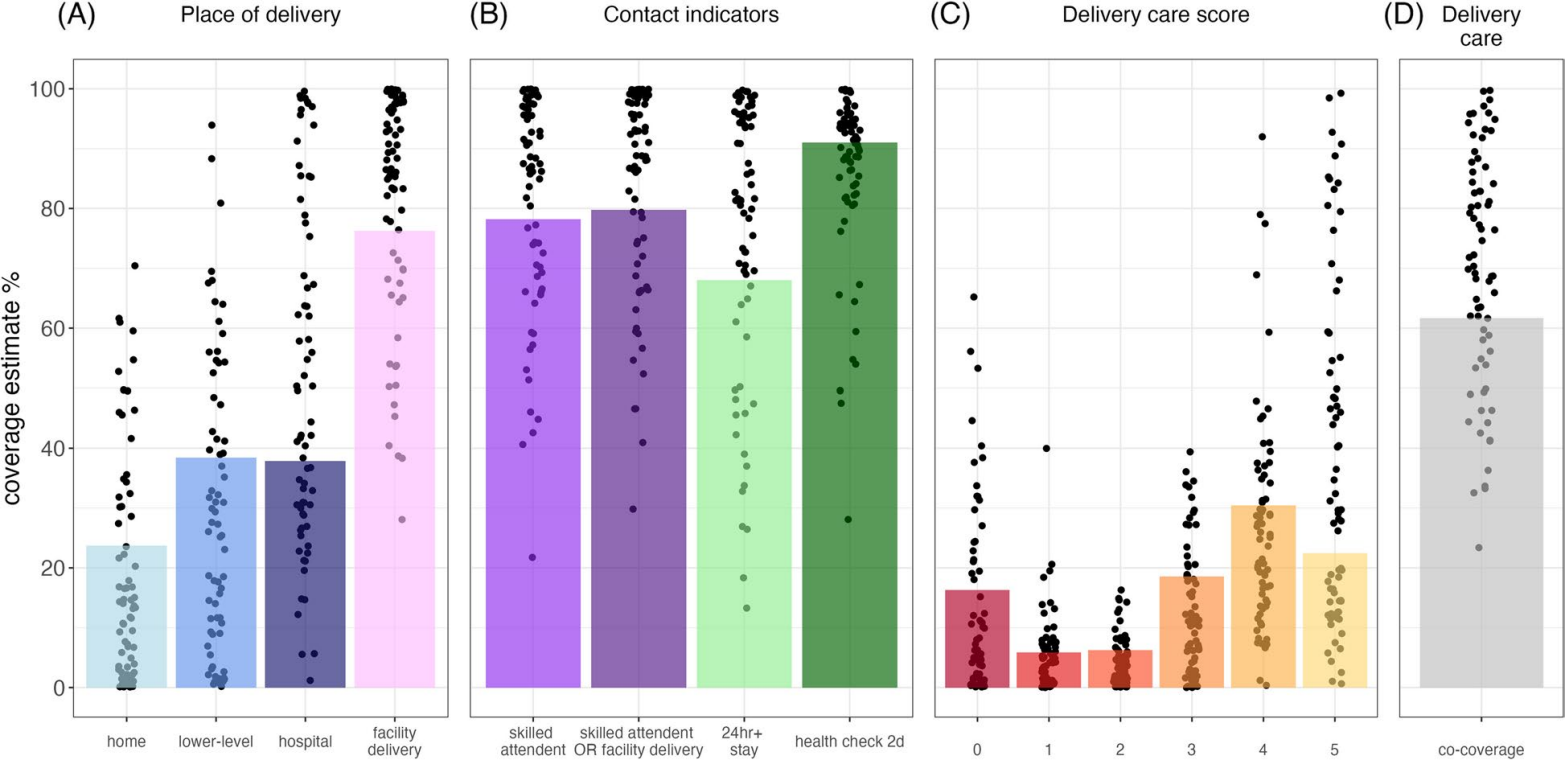
Country (dot) and pooled sample (bar) coverage of place of delivery (**A**), skilled attendant at birth and postnatal care services (**B**), delivery care scores (**C**), and overall delivery care indicator (**D**)

Figure 1 shows the country-level estimates for each indicator, delivery care score, and the overall delivery care indicator, overlaid with the mean estimate of the pooled sample. Mean facility delivery coverage across all countries was 76% split evenly between lower-level facilities (38%) and hospitals (38%) (Fig. 1A). The pooled sample estimate for skilled attendant at birth was 78%, hospital stay of 24h + was 68%, and postnatal care within 48 h was 91% (Fig. 1B).

**Fig. 2:**
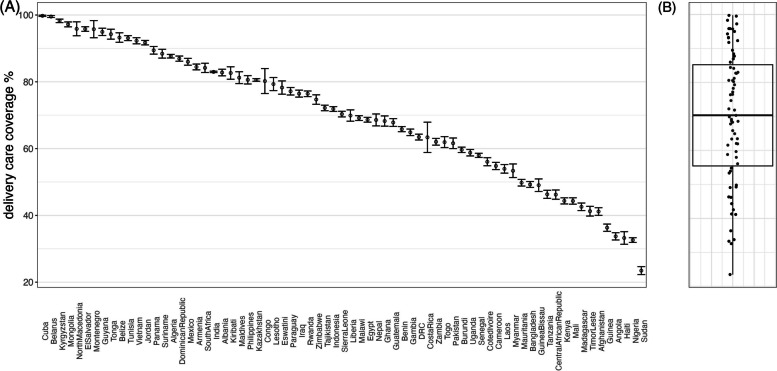
Proportion of women who received delivery care for their most recent live birth in each country. Error bars indicate 95% CIs (**A**). Panel **B** shows delivery care coverage estimates for all countries overlayed with median and interquartile range

Figure 1C shows the distribution of delivery care scores, or the proportion of births in each country assigned to each delivery score. The sum of the coverage estimates in columns 0, 1, 2, 3, 4, and 5 total 100% for each country. This distribution is skewed to the left, with 22.5% of women across all countries having received all interventions and scoring 5 out of 5; 30.5% of women achieved a score of 4; 18.6% had a score of 3 and 6% had a score of 2 or 1. About 16.3% did not receive any intervention. The delivery care coverage in the pooled sample was 61.7% (Fig. 1D). Country estimates for delivery care coverage ranged from 23.5% in Sudan to 100% in Cuba (Fig. 2A). The median delivery care score was 70.3% [IQR: 55.4 – 85.2%] (Fig. 2B). Country estimates and confidence intervals are provided for each indicator in Supplementary Table 1.

**Fig. 3:**
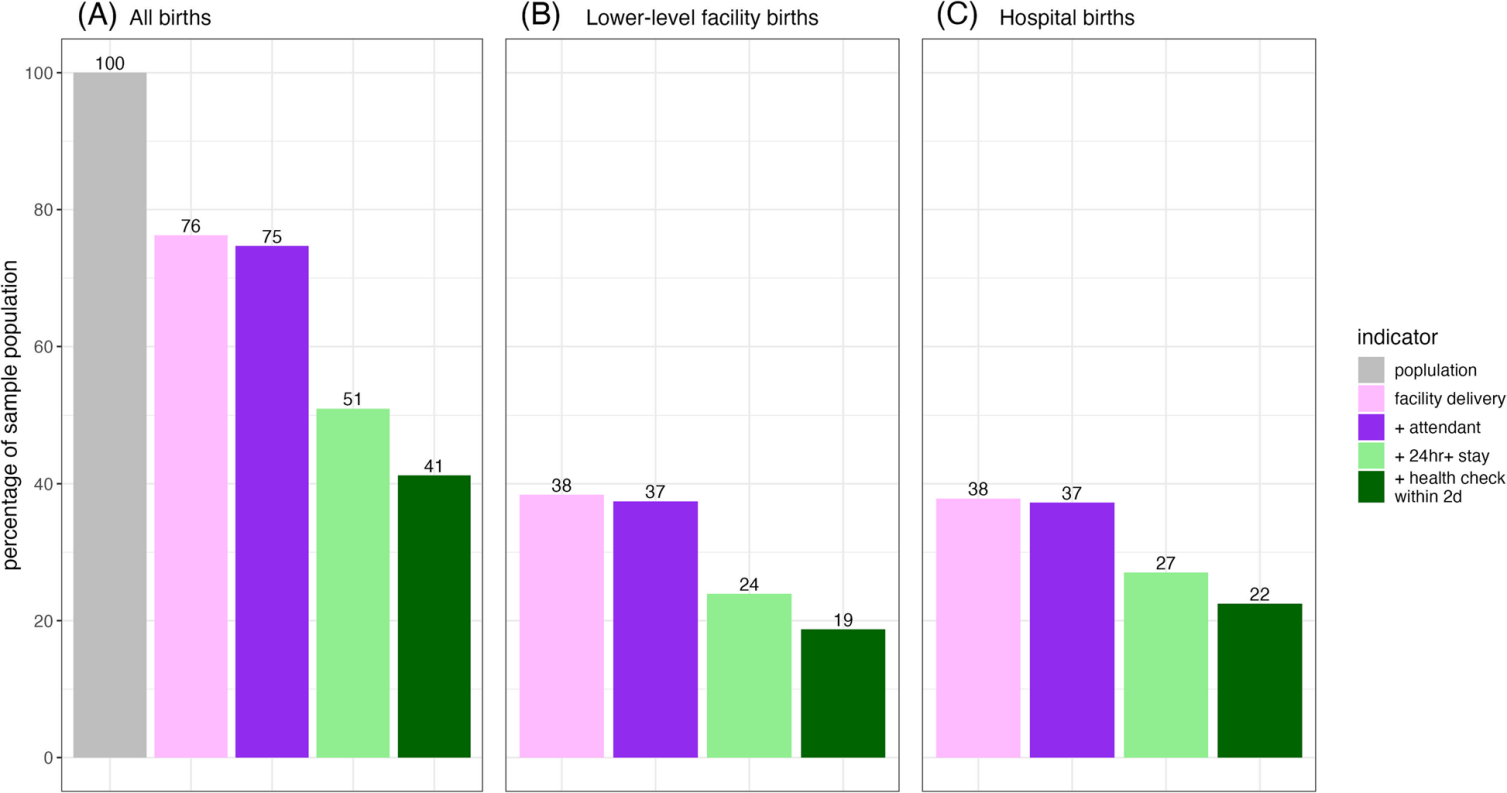
Delivery care cascade using pooled data weighted inversely proportional to country sample size (**A**) and stratified by births in lower-level facilities (**B**) and hospitals (**C**). Bars represent proportion of individuals who received each intervention in addition to intervention(s) to the left

Figure 3 shows the coverage cascade, starting with the pooled sample in the grey column on the far left. About three-quarters (76%) of women in the pooled sample had a facility delivery. Almost all the women who had a facility delivery also had a skilled attendant at delivery (75%). Almost one-fourth of the original sample was lost when 24h + facility stay was included. Ten percent of the pooled sample who had the first three interventions did not receive a health check within 2 days of delivery. Less than half of all women in this study (41%) received all 4 interventions, resulting in a gap of 35 percentage points compared to facility delivery coverage. The cascade drop-off pattern in both lower-level facility births (Fig. 3B) and hospital births (Fig. 3C) is similar to the pooled sample (Fig. 3A).

**Fig. 4:**
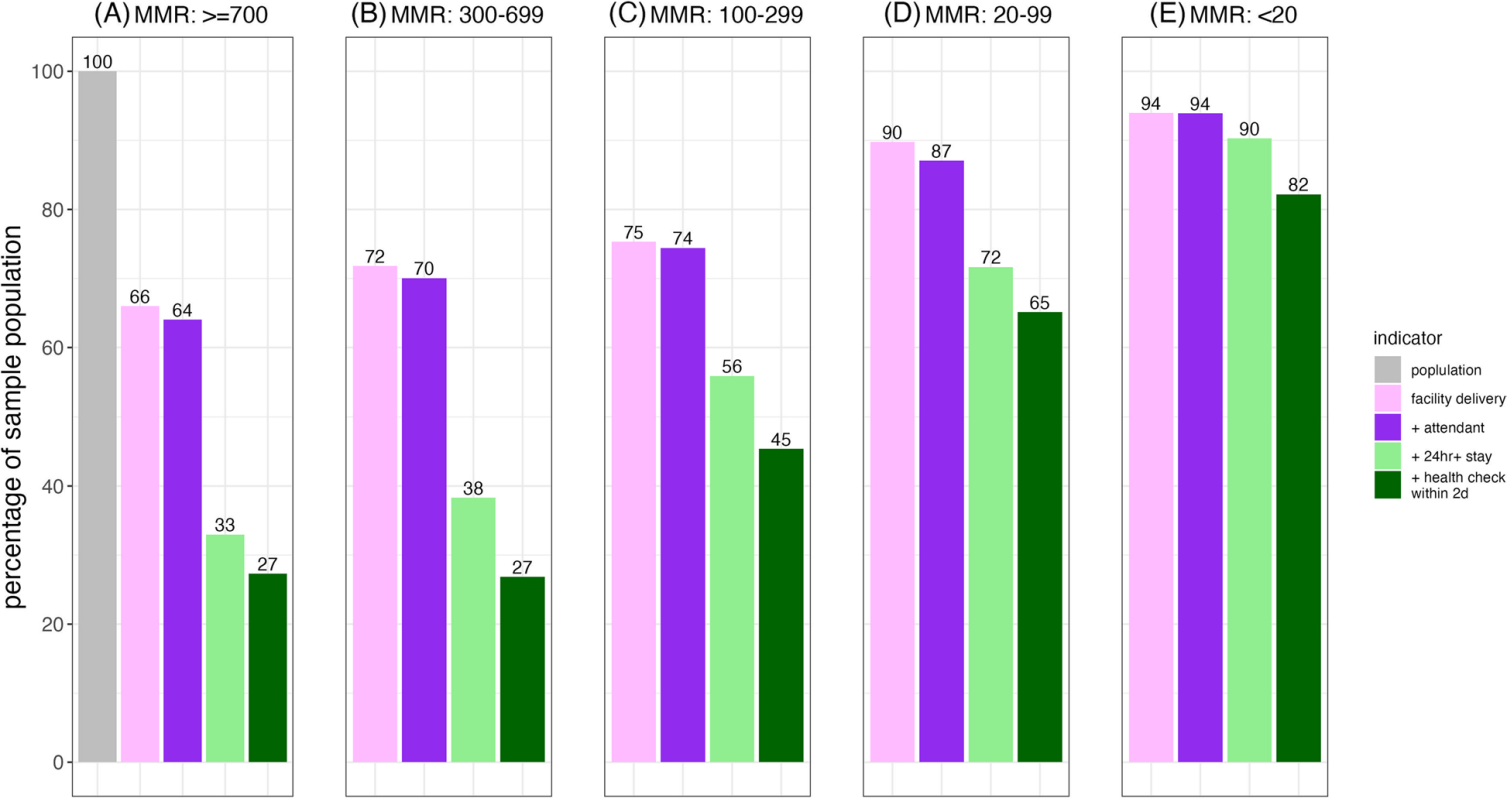
Delivery care cascade using pooled data stratified by maternal mortality ratio phases: greater than or equal to 700 (**A**), 300 to 699 (**B**), 100 to 299 (**C**), 20 to 99 (**D**), less than 20 (**E**)

The Fig. 4 cascades stratify the total pooled sample by MMR phase based on the transition framework proposed by Boerma et al. [26]: greater than or equal to 700, 300–699, 100–299, 20–99, and less than 20 maternal deaths per 100,000 live births. In each cascade, the percentage of women receiving additional interventions increase as the mortality ratio decreases. For example, the proportion of women who received all delivery care interventions is 27% where MMR is highest and second highest. Less than half (45%) of the women in the middle MMR phase, over half (65%) of the women where MMR is second lowest, and over three-fourths (82%) of women where MMR is lowest received all delivery care interventions.

**Fig. 5:**
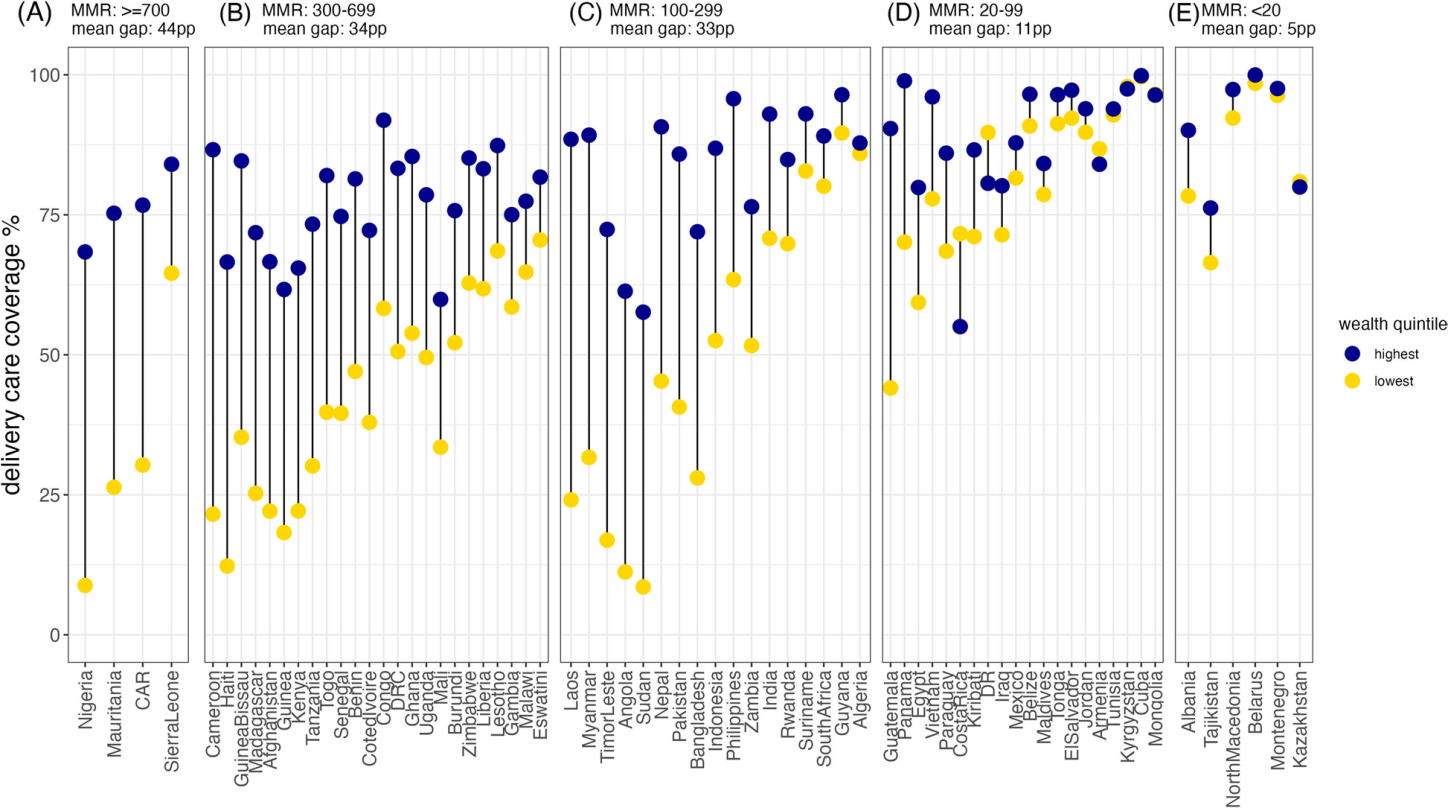
Mean delivery care coverage gap, between highest and lowest wealth quintile, among countries in each mortality transition phase: greater than or equal to 700 (**A**), 300 to 699 (**B**), 100 to 299 (**C**), 20 to 99 (**D**), less than 20 (**E**). Cascades for each country by MMR phase also show that as MMR goes down, the percentage of women who are getting all four indicators goes up (Supplementary Figure 1)

Figure 5 shows delivery care in each country, by both MMR phase and wealth quintile. The average absolute difference between highest and lowest wealth quintile goes down as MMR goes down. Where MMR is highest, the average difference in delivery and postnatal care between wealthiest and poorest quintiles is 44pp (Fig. 5A). In the panels where MMR is second and third highest, the difference in delivery care is 34pp and 33pp (Fig. 5B and C). In the panel where MMR is second lowest, the average delivery care difference is 11pp (Fig. 5D), and where MMR is lowest, the delivery care difference is 5pp (Fig. 5E).

There is a coverage gap between facility delivery and delivery care in most countries (Supplementary Figure 2). Women who experience a facility delivery do not receive at least some of the other recommended delivery interventions in countries where facility delivery is higher than delivery care coverage. In Madagascar and Myanmar many women had home births which were attended by skilled personnel, had a health check within 2 days, or both, resulting in facility delivery coverage which is lower than delivery care coverage. The average mean difference between facility delivery coverage and delivery care coverage ranges from 8pp where MMR is second lowest (Supplementary Figure 2D), to 15pp where MMR is second highest (Supplementary Figure 2B).

There is a negative linear trend between maternal mortality ratio and delivery care coverage (Supplementary Figure 3). MMR decreases by about 80 deaths per 100,000 live births for every 10% improvement in delivery care coverage based on the linear model. However, r^2^ is only 0.37.

## Discussion

Many countries show a gap between facility delivery and delivery care coverage indicating that many mothers are not receiving all recommended services when they give birth. Women tend to receive higher delivery care coverage, and experience smaller gaps between the highest and lowest wealth quintiles in countries where MMR is lower.

This study highlights where women miss opportunities to receive delivery care. Facility delivery, even as the comparative standard in this study, is a place of missed opportunities with about 25% of women delivering at home. A recent study from Nigeria and Ethiopia found that a large proportion of women deemed facility delivery unnecessary [28]. Rural women reported that facilities were too far away, or they had no transport, and urban women described barriers to getting permission and paying costs. Results of this study also show a gap in coverage between those who only deliver in a facility with a skilled attendant and those who also stay at the facility for at least 24 h. Many factors may influence why a woman did not stay in a facility for at least 24 h postpartum. In some cases, these women may have lived closer to the facility and experienced no complications, or they may have delivered in a facility with limited physical space for an overnight stay [29]. Neonatal size, gestational age, and mode of delivery are additional factors which influence duration of postpartum stay [30]. It will be necessary to contextualize the service populations of each delivery facility to improve the coverage and quality of postpartum stay.

Delivery care coverage ranks a hospital delivery as preferable to a lower-level facility delivery. This assumes hospitals are better equipped with emergency obstetric and newborn care (EmONC) and therefore have a better chance of treating obstetric complications which lead to maternal death [31]. There is evidence to support this. A recent study found that childbirth service readiness was higher for hospitals than health centers and clinics which had both lower and more variable readiness [32]. Another study found readiness varied by facility type, with hospitals more ready to care for small and sick newborns than other health facilities [33].

To address maternal health challenges in the SDG era countries should invest in improving health system capacity, including coverage of both routine reproductive health care and more advanced obstetric care [34]. Boerma and colleagues argue that both hospital and lower-level facilities play a crucial role in transitioning countries out of high MMR, into lower MMR phases. The transition out of high-mortality phases has involved a large increase in institutional births in lower-level facilities, while subsequent progress has been characterized by rapid increases in hospital births [26].

The average annual rate of reduction (AAR) in global MMR from 2000 to 2020 was 2.1% but achieving the 2030 MMR SDG target of 70 will require an unprecedented 11.6% AAR in the coming years [24]. Dramatic and rapid MMR reduction will require health system capacity improvements from all countries. Additionally, the gaps we observe go beyond a need to improve delivery care to prevent direct obstetric causes. Other factors, which put sub-populations at greater risk of maternal mortality, need to be addressed such as harmful gender norms resulting in low prioritization of sexual and reproductive health services for women and girls [1].

There were a few limitations to this study, which should be mentioned. Given country-to-country variability there was no way to know the precise level of care a woman received at each place of delivery in the facility level categorization, and women may have been referred to higher level facilities due to health complications which were not necessarily met with higher quality care. There is the potential for recall bias surrounding details such as the timing of postnatal care. There are significant challenges to estimating MMR: confidence intervals may be wide, adjustments are needed to address underreporting and misclassification, and countries without reliable data rely entirely on model-based estimates [35]. Finally, we use women’s responses to combine delivery and postnatal care interventions as a summary of care, although we do not measure quality of care on an individual level.

## Conclusions

Maternal health measures that capture the quality-of-care women receive during birth and the postnatal period are vital to understanding and reducing mortality. Coverage levels have improved and as data on the quality of maternal health interventions become available it will be possible to measure health gains more precisely. However, delivery care gaps exist across countries, wealth quintiles, and MMR phases. Aligning global priorities with the resources required to maintain effective facility-based care presents enormous challenges. Additional studies are needed to measure content of the specific interventions women are receiving, facility readiness at the point of care, and sub-national variation in quality of delivery care. It will also be necessary to understand mortality drivers within specific contexts, so that limited resources may be deployed where they will have the most impact on reducing maternal mortality.

### Supplementary Information

Supplementary Material 1.

Supplementary Material 2.

Supplementary Material 3.

Supplementary Material 4.

Supplementary Material 5.

Supplementary Material 6.

## Data Availability

The household survey data that support the findings of this study are publicly available. All analysis code is publicly available on the Countdown to 2030 Github repository: https://github.com/countdownto2030/delivery_care_coverage_gaps.
