# Supplementary material for: Delivery and postnatal care among women in 71 low- and middle-income countries: analyzing coverage gaps using household surveys
Source: BMC Pregnancy Childbirth. 2024 Jul 26;24:505. doi: 10.1186/s12884-024-06681-y (PMC11282627; doi:10.1186/s12884-024-06681-y)
Supplement: Supplementary file 4 — Supplementary Material 4. [file 12884_2024_6681_MOESM4_ESM.docx]

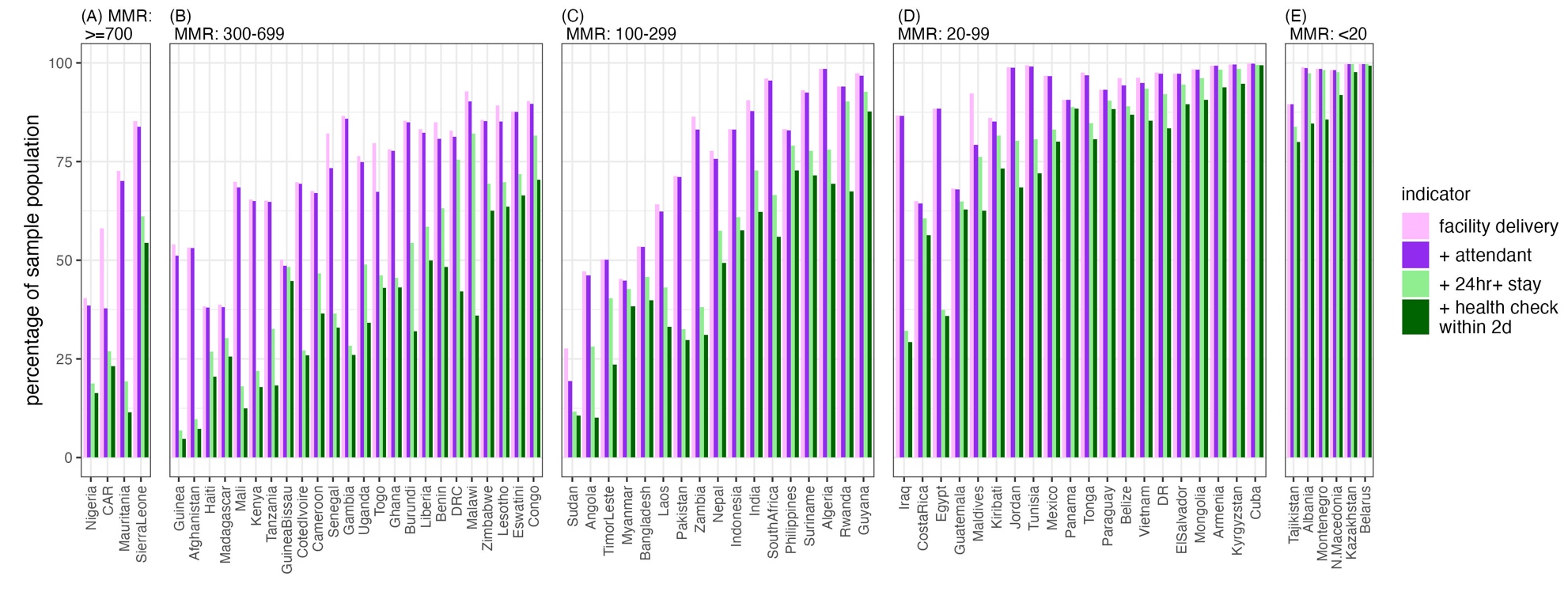


Supplementary Figure 1. Delivery care cascade using country data stratified by maternal mortality ratio transition phases: greater than or equal to 700 (A), 300 to 699 (B), 100 to 299 (C), 20 to 99 (D), less than 20 (E).
