# Supplementary material for: Delivery and postnatal care among women in 71 low- and middle-income countries: analyzing coverage gaps using household surveys
Source: BMC Pregnancy Childbirth. 2024 Jul 26;24:505. doi: 10.1186/s12884-024-06681-y (PMC11282627; doi:10.1186/s12884-024-06681-y)
Supplement: Supplementary file 6 — Supplementary Material 6. [file 12884_2024_6681_MOESM6_ESM.docx]

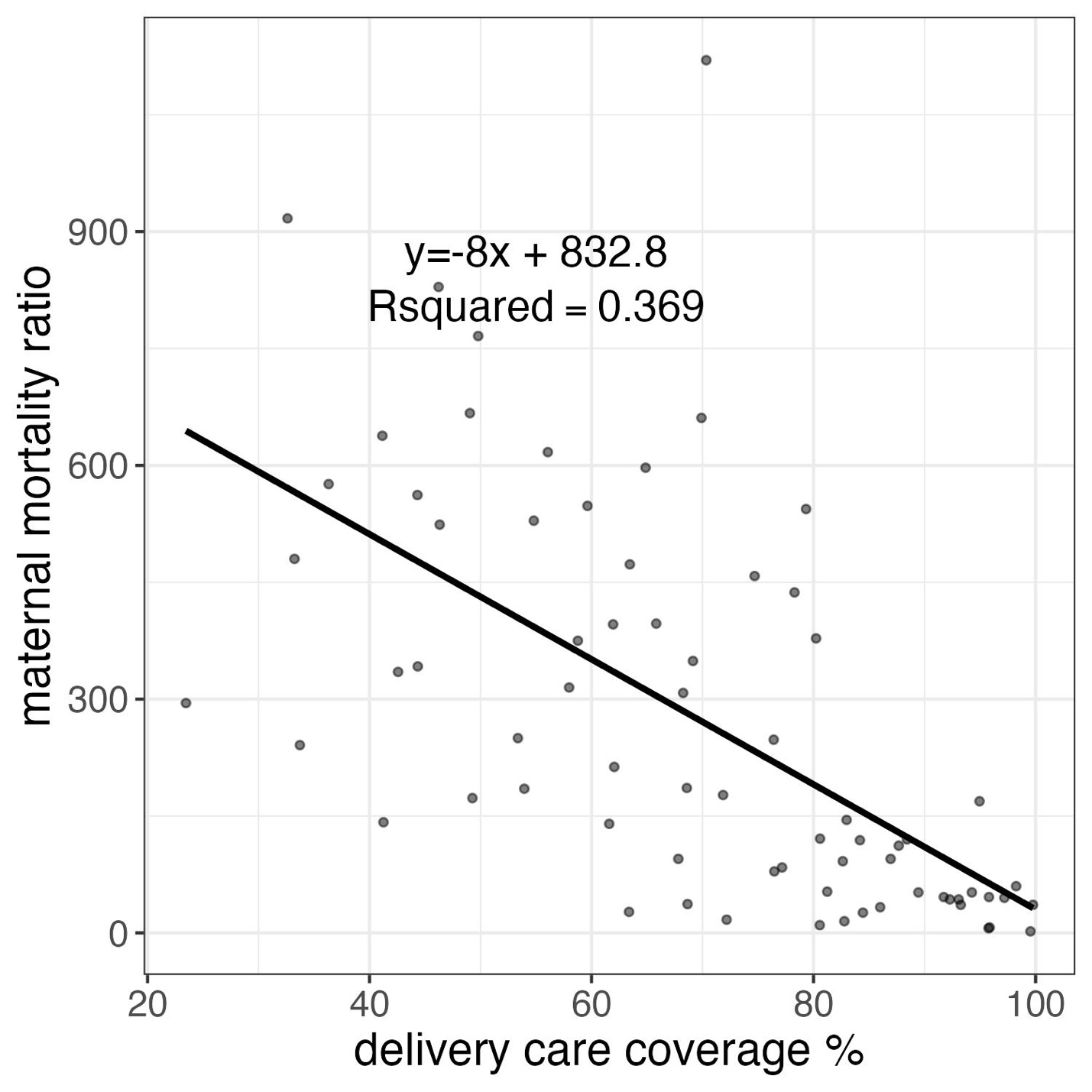


Supplementary Figure 3. Linear trend between the maternal mortality ratio and delivery care coverage among all countries in the study.
